# Associated factors and gender differences of falls in older adults with hypertension: a national cross-sectional survey

**DOI:** 10.3389/fpubh.2025.1537587

**Published:** 2025-04-16

**Authors:** Yazhu Wang, Yingying Zhang, Shiwei Cao, Xiyu Chen, Xiaobing Xian, Tengfei Niu

**Affiliations:** ^1^Department of Cardiology, The Shapingba Hospital, Chongqing University (People’s Hospital of Shapingba District), Chongqing, China; ^2^The Second Clinical College, Chongqing Medical University, Chongqing, China; ^3^The First Clinical College, Chongqing Medical University, Chongqing, China; ^4^The Thirteenth People’s Hospital of Chongqing, Chongqing, China; ^5^Chongqing Geriatrics Hospital, Chongqing, China; ^6^Department of Basic Courses, Chongqing Medical and Pharmaceutical College, Chongqing, China

**Keywords:** hypertension, falls, older adults, CLHLS, health ecology model

## Abstract

**Background:**

Falls have become a crucial public health problem among older adults, especially those with hypertension. However, the current understanding of the risk of falls among them is still insufficient. The purpose of this study was to investigate the factors associated with falls and their gender differences among older adults with hypertension in China.

**Methods:**

Based on the cross-sectional data of the Chinese Longitudinal Healthy Longevity Survey (CLHLS) 2018 database, this study defined 24 possible associated factors based on the five dimensions of the Health Ecology Model. Binary Logistic Regression Model was used to analyze the impact of each factor on falls among older adults with hypertension.

**Results:**

The prevalence rate of falls in older adults with hypertension in China was 22.60%. Falls are associated with a variety of factors. Specifically, gender, self-rated health, hearing impairment, stroke, instrumental activities of daily living (IADL) disability, basic activities of daily living (BADL) disability, exercise, fresh fruit and taste preference are significant associated factors for falls among older adults with hypertension. Among them, the effects of self-rated health, stroke and exercise on falls are only significant in female with hypertension. The effect of fresh fruit on falls was significant only in men with hypertension.

**Conclusion:**

The findings highlight that the current situation of falls among older adults with hypertension requires attention, necessitating comprehensive measures for prevention and control.

## 1 Introduction

As indicated by the latest data released by the World Health Organization (WHO) in 2023, around 1.28 billion adults worldwide suffer from hypertension. The number of patients with hypertension in China has reached 270 million, and its prevalence continues to rise (1). As a common chronic disease, hypertension causes mental illness and other chronic diseases that increase the burden on the body, so people with hypertension may have a higher risk of falling (2–4). In addition, antihypertensive drugs, as the most commonly prescribed drugs for older adults with hypertension, often cause orthostatic hypotension and then fall in older adults (5). A recent study suggested that high blood pressure medications enhanced the risk of harmful falls by 30 to 40 percent (4). Hence, older adults with hypertension have a higher chance of falling than the average people, and the consequences of falls can be more severe (6). This suggests that a more informed perspective on the epidemiological characteristics and associated factors of falls in hypertensive patients can help identify individuals at high risk of falls as early as possible, thereby improving their life quality. According to previous surveys, in excess of 25% of older adults worldwide encounter falls every year. About 35% of older adults who live in the community experience at least one fall every year, while this number rises to 50% among those in long-term care (7, 8). Falls in older people can also result in a drop in their life quality, loss of self-care, disability, and even death (9). These places a huge burden on social services and health care systems. In recent years, there has been heightened attention to early diagnosis and prevention of illness or injury. A multitude of previous studies have reported the factors associated with falls among older adults. For example, a study by Haibin Zhou et al. suggested that disease, living alone, and impaired vision are risk factors for falls among older adults in the community (10). Xingxing Xian et al. also emphasized that fall prevention programs might need to prioritize behavior-related risk factors based on WHO’s Risk Factor Model for Falls (11). In addition, some studies have highlighted that there might be some gender differences in the incidence of falls among older adults (12), this may be because sex hormones can affect muscle atrophy, which can result in an unsteady gait (13). Undoubtedly, the findings of these previous studies have made outstanding contributions to the prevention and improvement of falls among older adults recently. However, these findings still might not be applied to older adults with hypertension in China.

Considering that the associated factors of falls among older adults with hypertension are numerous and complex, we introduced the Health Ecology Model (HEM) in this study, as it is often used by scholars to understand these complex effects (14, 15). HEM emphasizes the multiple levels of environmental and individual influences and the complexity of influencing factors. It studies the associated factors of diseases from five dimensions: personal characteristics, behavioral characteristics, interpersonal relationships, living and working conditions and policy environment. It is an important theoretical model to guide the field of public health and solve population health problems.

In summary, the purpose of this study was to use the cross-sectional data published in the Chinese Longitudinal Healthy Longevity Survey (CLHLS) in 2018, define possible associated factors based on the five dimensions of HEM, and analyze the associated factors and gender differences of falls among older adults with hypertension in China with Logistic Regression Model. These findings can help healthcare professionals better understand the needs of older adults with hypertension to provide accurate care and health management programs, and provide important references for policymakers to formulate relevant intervention strategies.

## 2 Method

### 2.1 Study population

The data for this study are derived from cross-sectional data published in 2018 by the CLHLS (16). The CLHLS baseline study began in 1998, and from 1998 to 2018, eight follow-up studies were conducted in 23 provinces, cities, and autonomous regions of China. The survey includes individual micro-data on family structure and living arrangements, marriage, health, and socioeconomic characteristics of older adults. All survey data for the project can be requested from the CLHLS website^1^. The CLHLS received ethical approval from the Bioethics Committee of Peking University (IRB00001052-13074), and each participant completed an informed consent form before data collection.

**Figure 1:**
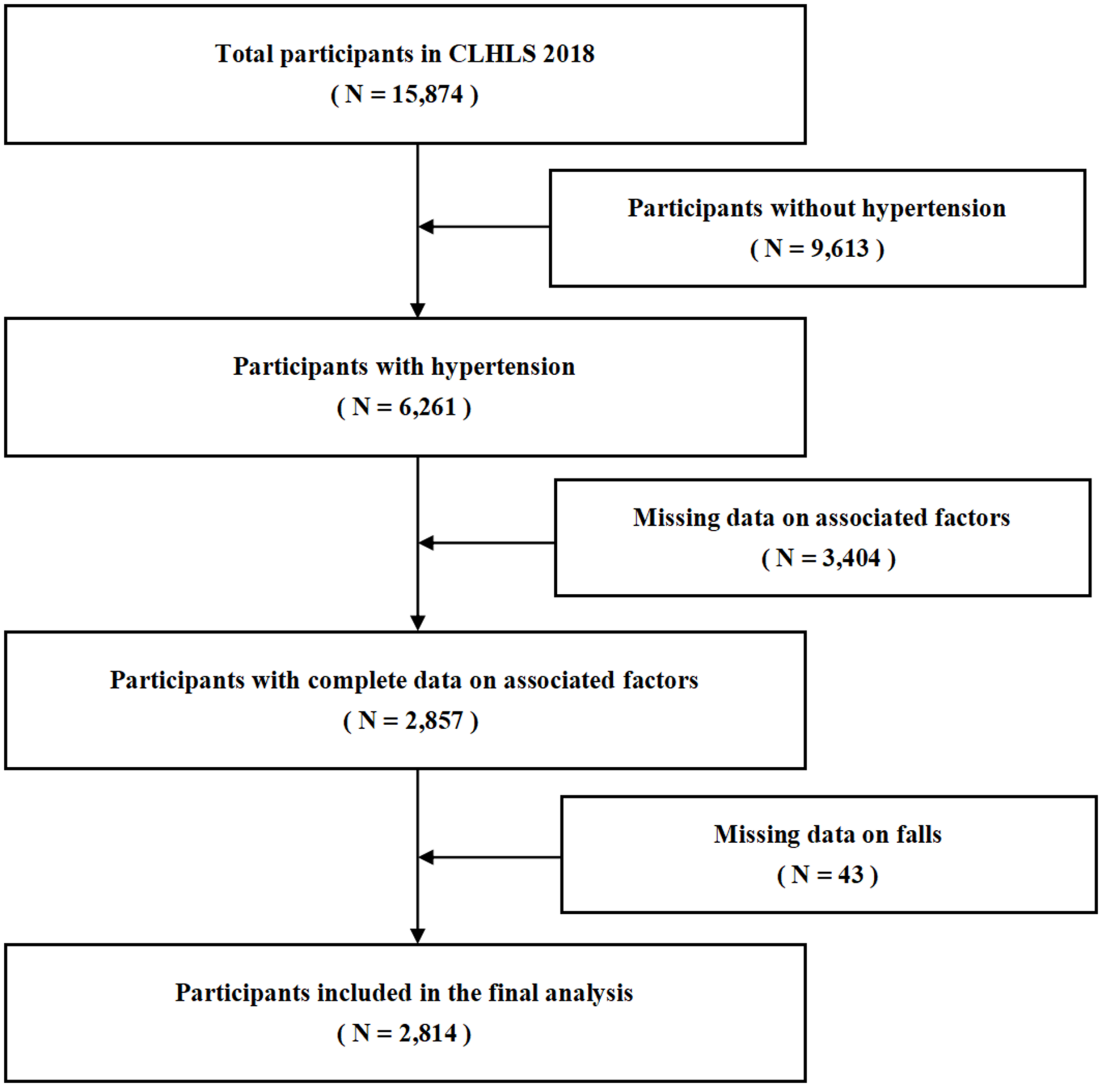
Study sample screening process.

The screening of the study population was based on the question in the questionnaire: “Have you been diagnosed with high blood pressure by a doctor?” Respondents who answered “yes” to this question were included in our study. In 2018, CLHLS surveyed 15,874 participants aged 65 and above, of whom 6,261 were diagnosed with hypertension by doctors. Subsequent exclusion of missing data on falls and influencing factor variables resulted in the inclusion of 2,814 participants in this analysis. The sample screening process is shown in Figure 1.

### 2.2 Falls

CLHLS contains questions that are directly related to fall events. All information was obtained through face-to-face interviews conducted by trained investigators or local doctors/nurses. Falls among older adults with hypertension were defined based on the question “Have you fallen in the past year?.” If the respondent answered “yes” to the question, it was defined as a fall. Previous studies have also shown the reliability of this measurement (17, 18).

### 2.3 Associated factors

Based on the five dimensions of the HEM (personal characteristics, behavioral characteristics, interpersonal relationships, living and working conditions and policy environment) (19), a total of 24 potential associated factors variables were defined from the CLHLS. The HEM emphasizes that health is influenced by both individual and environmental factors. Collecting and organizing variables based on the HEM allows us to include more comprehensive and structured variables. Specifically, in the personal characteristics dimension, variables include gender, age, body mass index (BMI), abdominal obesity, self-rated health, hearing impairment, visual impairment, heart disease, diabetes, stroke, basic activities of daily living (BADL) disability, and instrumental activities of daily living (IADL) disability. BMI is calculated by dividing your weight (in kilograms) by your height (in meters) squared. Abdominal obesity is defined as a waist circumference superior to 102 cm in men and 88 cm in women (20). The Basic Activities of Daily Living (BADL) Scale and the Instrumental Activities of Daily Living (IADL) Scale were used to measure the ability to perform daily activities among older adults, and participants were considered to be free of functional impairment (21) when they were all defined as “able to complete independently” on either the BADL or IADL Scales.

Behavioral characteristics dimension includes exercise, smoking, drinking, self-reported quality of life, fresh fruit, grease, and taste preference.

The interpersonal relationships dimension includes co-residence, residence, and marital status.

The dimension of living and working conditions contains education level.

The policy environment dimension includes insurance.

Detailed variable measurements and assignments are shown in Appendix 1.

### 2.4 Statistical analysis

Categorical variables are described in frequency and percentage [*n* (%)]. The Chi-square test is used to compare the differences between different demographic characteristics in whether falls occur. Multifactor logistic regression was used to analyze the relationship between various associated factors and falls. In addition, we took falls as the dependent variable and gender and other associated factors as covariates, and conducted a product interaction analysis to explore whether there are gender differences in the associated factors of falls. We report odds ratios (OR) and 95% confidence intervals (CI) for all variables. All statistical analyses were performed using SPSS 25.0 software. The statistical test was a bilateral test, and a *p* value less than 0.05 indicated significant statistical significance.

## 3 Result

### 3.1 Results of descriptive analysis

According to the statistical results, 636 (22.60%) of the respondents experienced falls. Of all the respondents, 1,670 (59.35%) were female, 1,303 (46.30%) were married, and 1,371 (48.72%) were illiterate. 1,603 (56.97%) respondents were over 80 years old, 1,591 (56.54%) rated their health as poor, 1,760 (62.54%) had IADL disability, and 2,562 (91.04%) preferred vegetable grease. Among the respondents, 1,966 (69.86%) had a light taste. 1,873 (66.56%) did not exercise, 2,409 (85.61%) did not smoke, 2,436 (86.57%) did not drink alcohol, 1,671 (59.38%) were not insured, and 769 (27.33%) had abdominal obesity (Table 1).

**Table 1:** Characteristics of participants.

Variables	Total (*n* = 2,814)	No falls (*n* = 2,178)	Falls (*n* = 636)	Statistic	*p*
Gender, *n* (%)				*χ*^2^ = 16.72	<0.001
Female	1,670 (59.35)	1,248 (57.30)	422 (66.35)		
Male	1,144 (40.65)	930 (42.70)	214 (33.65)		
Age, *n* (%)				*χ*^2^ = 15.83	<0.001
60–79	1,211 (43.03)	981 (45.04)	230 (36.16)		
≥ 80	1,603 (56.97)	1,197 (54.96)	406 (63.84)		
BMI, *n* (%)				*χ*^2^ = 10.43	0.015
< 18.5	306 (10.87)	231 (10.61)	75 (11.79)		
18.5–24	1,358 (48.26)	1,022 (46.92)	336 (52.83)		
24–28	816 (29.00)	659 (30.26)	157 (24.69)		
≥ 28	334 (11.87)	266 (12.21)	68 (10.69)		
Abdominal obesity, *n* (%)				*χ*^2^ = 0.69	0.407
No	2,045 (72.67)	1,591 (73.05)	454 (71.38)		
Yes	769 (27.33)	587 (26.95)	182 (28.62)		
Self-rated health, *n* (%)				*χ*^2^ = 24.48	<0.001
Good	1,223 (43.46)	1,001 (45.96)	222 (34.91)		
Bad	1,591 (56.54)	1,177 (54.04)	414 (65.09)		
Hearing impairment, *n* (%)				*χ*^2^ = 27.81	<0.001
No	1,860 (66.10)	1,495 (68.64)	365 (57.39)		
Yes	954 (33.90)	683 (31.36)	271 (42.61)		
Visual impairment, *n* (%)				*χ*^2^ = 5.93	0.015
No	1,938 (68.87)	1,525 (70.02)	413 (64.94)		
Yes	876 (31.13)	653 (29.98)	223 (35.06)		
Heart disease, *n* (%)				*χ*^2^ = 4.81	0.028
No	2,254 (80.10)	1,764 (80.99)	490 (77.04)		
Yes	560 (19.90)	414 (19.01)	146 (22.96)		
Diabetes, *n* (%)				*χ*^2^ = 0.50	0.481
No	2,435 (86.53)	1,890 (86.78)	545 (85.69)		
Yes	379 (13.47)	288 (13.22)	91 (14.31)		
Stroke, *n* (%)				*χ*^2^ = 12.59	<0.001
No	2,441 (86.74)	1,916 (87.97)	525 (82.55)		
Yes	373 (13.26)	262 (12.03)	111 (17.45)		
BADL disability, *n* (%)				*χ*^2^ = 36.93	<0.001
No	2,301 (81.77)	1,833 (84.16)	468 (73.58)		
Yes	513 (18.23)	345 (15.84)	168 (26.42)		
IADL disability, *n* (%)				*χ*^2^ = 55.80	<0.001
No	1,054 (37.46)	896 (41.14)	158 (24.84)		
Yes	1,760 (62.54)	1,282 (58.86)	478 (75.16)		
Exercise, *n* (%)				*χ*^2^ = 0.85	0.355
No	1,873 (66.56)	1,440 (66.12)	433 (68.08)		
Yes	941 (33.44)	738 (33.88)	203 (31.92)		
Smoking, *n* (%)				*χ*^2^ = 6.29	0.012
No	2,409 (85.61)	1845 (84.71)	564 (88.68)		
Yes	405 (14.39)	333 (15.29)	72 (11.32)		
Drinking, *n* (%)				*χ*^2^ = 2.70	0.100
No	2,436 (86.57)	1,873 (86.00)	563 (88.52)		
Yes	378 (13.43)	305 (14.00)	73 (11.48)		
Self-reported quality of life, *n* (%)				*χ*^2^ = 5.30	0.021
Good	1,992 (70.79)	1,565 (71.85)	427 (67.14)		
Bad	822 (29.21)	613 (28.15)	209 (32.86)		
Fresh fruit, *n* (%)				*χ*^2^ = 12.71	<0.001
No	719 (25.55)	522 (23.97)	197 (30.97)		
Yes	2,095 (74.45)	1,656 (76.03)	439 (69.03)		
Grease, *n* (%)				*χ*^2^ = 0.91	0.340
Animal grease	252 (8.96)	189 (8.68)	63 (9.91)		
Vegetable grease	2,562 (91.04)	1,989 (91.32)	573 (90.09)		
Taste preference, *n* (%)				*χ*^2^ = 5.72	0.017
Other	848 (30.14)	632 (29.02)	216 (33.96)		
Light taste	1,966 (69.86)	1,546 (70.98)	420 (66.04)		
Co-residence, *n* (%)				*χ*^2^ = 0.87	0.351
Alone	495 (17.59)	391 (17.95)	104 (16.35)		
Other	2,319 (82.41)	1,787 (82.05)	532 (83.65)		
Residence, *n* (%)				*χ*^2^ = 0.00	0.960
Urban	1,378 (48.97)	1,066 (48.94)	312 (49.06)		
Rural	1,436 (51.03)	1,112 (51.06)	324 (50.94)		
Marital status, *n* (%)				*χ*^2^ = 12.11	<0.001
Other	1,511 (53.70)	1,131 (51.93)	380 (59.75)		
Married	1,303 (46.30)	1,047 (48.07)	256 (40.25)		
Education level, *n* (%)				*χ*^2^ = 21.47	<0.001
Illiteracy	1,371 (48.72)	1,012 (46.46)	359 (56.45)		
Primary school or below	1,060 (37.67)	847 (38.89)	213 (33.49)		
Secondary school or above	383 (13.61)	319 (14.65)	64 (10.06)		
Insurance, *n* (%)				*χ*^2^ = 0.24	0.625
No	1,671 (59.38)	1,288 (59.14)	383 (60.22)		
Yes	1,143 (40.62)	890 (40.86)	253 (39.78)		

*χ2, Chi-square test; BMI, body mass index; IADL, instrumental activity of daily living; BADL, basic activity of daily living.*

The frequency of falls in hypertensive patients was significantly different in age, gender, BMI, self-rated health, hearing impairment, visual impairment, heart disease, stroke, IADL disability, BADL disability, smoking, fresh fruit, taste preference, self-reported quality of life, marital status, and education level (*p* < 0.05). Detailed descriptive results are presented in Table 1.

### 3.2 Associated factors of falls among older adults with hypertension

The findings of multivariate logistic regression indicated that gender, self-rated health, hearing impairment, stroke, BADL disability, IADL disability, exercise, fresh fruit, and taste preference were significant risk factors for falls in older patients with hypertension (*p* < 0.05). Males (OR = 0.766; 95% CI: 0.602–0.975) were less likely to fall than females, and those who self-rated to be in poor health (OR = 1.376; 95% CI: 1.116–1.695) were more likely to fall than those who self-rated to be in good health. Those with hearing impairment (OR = 1.274; 95% CI: 1.034–1.570), those with stroke (OR = 1.404; 95% CI: 1.088–1.811), those with BADL disability (OR = 1.367; 95% CI: 1.072–1.743), and those with IADL disability (OR = 1.625; 95% CI: 1.266–2.086) had a higher risk of falling than normal people, and people who exercised regularly (OR = 1.269; 95% CI: 1.029–1.566) were more likely to fall than those who did not. People who ate fresh fruit (OR = 0.775; 95% CI: 0.632–0.950) were less likely to fall than those who did not, and those with light taste (OR = 0.748; 95% CI: 0.615–0.909) were less likely to fall than those with other tastes (Table 2).

**Table 2:** Logistic regression results of falls.

Variables	OR	95% CI	*p*
Gender, *n* (%)			
Female	1.00 (Ref.)	1.00 (Ref.)	
Male	0.766	0.602–0.975	0.030
Age, *n* (%)			
60–79	1.00 (Ref.)	1.00 (Ref.)	
≥ 80	0.922	0.718–1.183	0.522
BMI, *n* (%)			
< 18.5	1.00 (Ref.)	1.00 (Ref.)	0.100
18.5–24	1.194	0.882–1.616	0.252
24–28	0.922	0.653–1.302	0.645
≥ 28	0.917	0.603–1.393	0.684
Abdominal obesity, *n* (%)			
No	1.00 (Ref.)	1.00 (Ref.)	
Yes	1.019	0.803–1.293	0.878
Self-rated health, *n* (%)			
Good	1.00 (Ref.)	1.00 (Ref.)	
Bad	1.376	1.116–1.695	0.003
Hearing impairment, *n* (%)			
No	1.00 (Ref.)	1.00 (Ref.)	
Yes	1.274	1.034–1.570	0.023
Visual impairment, *n* (%)			
No	1.00 (Ref.)	1.00 (Ref.)	
Yes	0.953	0.773–1.174	0.650
Heart disease, *n* (%)			
No	1.00 (Ref.)	1.00 (Ref.)	
Yes	1.139	0.907–1.430	0.264
Diabetes, *n* (%)			
No	1.00 (Ref.)	1.00 (Ref.)	
Yes	1.059	0.809–1.388	0.676
Stroke, *n* (%)			
No	1.00 (Ref.)	1.00 (Ref.)	
Yes	1.404	1.088–1.811	0.009
BADL disability, *n* (%)			
No	1.00 (Ref.)	1.00 (Ref.)	
Yes	1.367	1.072–1.743	0.012
IADL disability, *n* (%)			
No	1.00 (Ref.)	1.00 (Ref.)	
Yes	1.625	1.266–2.086	<0.001
Exercise, *n* (%)			
No	1.00 (Ref.)	1.00 (Ref.)	
Yes	1.269	1.029–1.566	0.026
Smoking, *n* (%)			
No	1.00 (Ref.)	1.00 (Ref.)	
Yes	0.798	0.587–1.084	0.148
Drinking, *n* (%)			
No	1.00 (Ref.)	1.00 (Ref.)	
Yes	1.195	0.883–1.617	0.248
Self-reported quality of life, *n* (%)			
Good	1.00 (Ref.)	1.00 (Ref.)	
Bad	1.084	0.877–1.341	0.455
Fresh fruit, *n* (%)			
No	1.00 (Ref.)	1.00 (Ref.)	
Yes	0.775	0.632–0.950	0.014
Grease, *n* (%)			
Animal grease	1.00 (Ref.)	1.00 (Ref.)	
Vegetable grease	0.825	0.603–1.128	0.227
Taste preference, *n* (%)			
Other	1.00 (Ref.)	1.00 (Ref.)	
Light taste	0.748	0.615–0.909	0.004
Co-residence, *n* (%)			
Alone	1.00 (Ref.)	1.00 (Ref.)	
Other	1.140	0.875–1.485	0.333
Residence, *n* (%)			
Urban	1.00 (Ref.)	1.00 (Ref.)	
Rural	0.971	0.807–1.169	0.758
Marital status, *n* (%)			
Other	1.00 (Ref.)	1.00 (Ref.)	
Married	0.942	0.746–1.190	0.618
Education level, *n* (%)			
Illiteracy	1.00 (Ref.)	1.00 (Ref.)	0.556
Primary school or below	0.925	0.739–1.158	0.497
Secondary school or above	0.831	0.588–1.174	0.293
Insurance, *n* (%)			
No	1.00 (Ref.)	1.00 (Ref.)	
Yes	0.979	0.811–1.182	0.823

*χ2, Chi-square test; BMI, body mass index; IADL, instrumental activity of daily living; BADL, basic activity of daily living; Ref., reference.*

### 3.3 Comparison of the associated factors of falls in older male and female patients with hypertension

Chi-square test results in Table 3 showed that the fall prevalence of older men with hypertension was significantly different in different BMI, self-rated health, hearing impairment, BADL disability, IADL disability, self-reported quality of life, fresh fruit, and education level (*p* < 0.05). The prevalence of falls in older women with hypertension was significantly different in different ages, BMI, self-rated health status, hearing impairment, stroke, BADL disability and IADL disability (*p* < 0.05).

**Table 3:** Chi-square test results of falls in male and female.

Variables	Male					Female				
	Total (*n* = 1,144)	No falls (*n* = 930)	Falls (*n* = 214)	Statistic	*p*	Total (*n* = 1,670)	No falls (*n* = 1,248)	Falls (*n* = 422)	Statistic	*p*
Age, *n* (%)				*χ*^2^ = 4.20	0.04				*χ*^2^ = 9.33	0.002
≥ 80	548 (47.90)	459 (49.35)	89 (41.59)			663 (39.70)	522 (41.83)	141 (33.41)		
≥ 80	596 (52.10)	471 (50.65)	125 (58.41)			1,007 (60.30)	726 (58.17)	281 (66.59)		
BMI, *n* (%)				*χ*^2^ = 6.11	0.107				*χ*^2^ = 6.01	0.111
< 18.5	106 (9.27)	88 (9.46)	18 (8.41)			200 (11.98)	143 (11.46)	57 (13.51)		
18.5–24	577 (50.44)	453 (48.71)	124 (57.94)			781 (46.77)	569 (45.59)	212 (50.24)		
24–28	339 (29.63)	287 (30.86)	52 (24.30)			477 (28.56)	372 (29.81)	105 (24.88)		
≥ 28	122 (10.66)	102 (10.97)	20 (9.35)			212 (12.69)	164 (13.14)	48 (11.37)		
Abdominal obesity, *n* (%)				*χ*^2^ = 0.11	0.736				*χ*^2^ = 0.71	0.401
No	1,049 (91.70)	854 (91.83)	195 (91.12)			996 (59.64)	737 (59.05)	259 (61.37)		
Yes	95 (8.30)	76 (8.17)	19 (8.88)			674 (40.36)	511 (40.95)	163 (38.63)		
Self-rated health, *n* (%)				*χ*^2^ = 10.78	0.001				*χ*^2^ = 12.44	<0.001
Good	527 (46.07)	450 (48.39)	77 (35.98)			696 (41.68)	551 (44.15)	145 (34.36)		
Bad	617 (53.93)	480 (51.61)	137 (64.02)			974 (58.32)	697 (55.85)	277 (65.64)		
Hearing impairment, *n* (%)				*χ*^2^ = 12.91	<0.001				*χ*^2^ = 14.78	<0.001
No	766 (66.96)	645 (69.35)	121 (56.54)			1,094 (65.51)	850 (68.11)	244 (57.82)		
Yes	378 (33.04)	285 (30.65)	93 (43.46)			576 (34.49)	398 (31.89)	178 (42.18)		
Visual impairment, *n* (%)				*χ*^2^ = 3.66	0.056				*χ*^2^ = 1.71	0.19
No	830 (72.55)	686 (73.76)	144 (67.29)			1,108 (66.35)	839 (67.23)	269 (63.74)		
Yes	314 (27.45)	244 (26.24)	70 (32.71)			562 (33.65)	409 (32.77)	153 (36.26)		
Heart disease, *n* (%)				*χ*^2^ = 2.68	0.102				*χ*^2^ = 1.60	0.206
No	947 (82.78)	778 (83.66)	169 (78.97)			1,307 (78.26)	986 (79.01)	321 (76.07)		
Yes	197 (17.22)	152 (16.34)	45 (21.03)			363 (21.74)	262 (20.99)	101 (23.93)		
Diabetes, *n* (%)				*χ*^2^ = 2.03	0.154				*χ*^2^ = 0.05	0.823
No	996 (87.06)	816 (87.74)	180 (84.11)			1,439 (86.17)	1,074 (86.06)	365 (86.49)		
Yes	148 (12.94)	114 (12.26)	34 (15.89)			231 (13.83)	174 (13.94)	57 (13.51)		
Stroke *n* (%)				*χ*^2^ = 5.92	0.015				*χ*^2^ = 8.00	0.005
No	975 (85.23)	804 (86.45)	171 (79.91)			1,466 (87.78)	1,112 (89.10)	354 (83.89)		
Yes	169 (14.77)	126 (13.55)	43 (20.09)			204 (12.22)	136 (10.90)	68 (16.11)		
BADL disability, *n* (%)				*χ*^2^ = 16.33	<0.001				*χ*^2^ = 17.23	<0.001
No	990 (86.54)	823 (88.49)	167 (78.04)			1,311 (78.50)	1,010 (80.93)	301 (71.33)		
Yes	154 (13.46)	107 (11.51)	47 (21.96)			359 (21.50)	238 (19.07)	121 (28.67)		
IADL disability, *n* (%)				*χ*^2^ = 23.76	<0.001				*χ*^2^ = 25.07	<0.001
No	535 (46.77)	467 (50.22)	68 (31.78)			519 (31.08)	429 (34.38)	90 (21.33)		
Yes	609 (53.23)	463 (49.78)	146 (68.22)			1,151 (68.92)	819 (65.62)	332 (78.67)		
Exercise, *n* (%)				*χ*^2^ = 2.59	0.108				*χ*^2^ = 0.41	0.522
No	693 (60.58)	553 (59.46)	140 (65.42)			1,180 (70.66)	887 (71.07)	293 (69.43)		
Yes	451 (39.42)	377 (40.54)	74 (34.58)			490 (29.34)	361 (28.93)	129 (30.57)		
Smoking, *n* (%)				*χ*^2^ = 0.51	0.474				χ^2^ = 1.35	0.245
No	811 (70.89)	655 (70.43)	156 (72.90)			1,598 (95.69)	1,190 (95.35)	408 (96.68)		
Yes	333 (29.11)	275 (29.57)	58 (27.10)			72 (4.31)	58 (4.65)	14 (3.32)		
Drinking, *n* (%)				*χ*^2^ = 0.21	0.648				*χ*^2^ = 0.03	0.852
No	852 (74.48)	690 (74.19)	162 (75.70)			1,584 (94.85)	1,183 (94.79)	401 (95.02)		
Yes	292 (25.52)	240 (25.81)	52 (24.30)			86 (5.15)	65 (5.21)	21 (4.98)		
Self-reported quality of life, *n* (%)				*χ*^2^ = 9.30	0.002				*χ*^2^ = 0.46	0.496
Good	799 (69.84)	668 (71.83)	131 (61.21)			1,193 (71.44)	897 (71.88)	296 (70.14)		
Bad	345 (30.16)	262 (28.17)	83 (38.79)			477 (28.56)	351 (28.12)	126 (29.86)		
Fresh fruit, *n* (%)				*χ*^2^ = 13.68	<0.001				*χ*^2^ = 3.14	0.076
No	302 (26.40)	224 (24.09)	78 (36.45)			417 (24.97)	298 (23.88)	119 (28.20)		
Yes	842 (73.60)	706 (75.91)	136 (63.55)			1,253 (75.03)	950 (76.12)	303 (71.80)		
Grease, *n* (%)				*χ*^2^ = 0.20	0.651				*χ*^2^ = 0.63	0.427
Animal grease	98 (8.57)	78 (8.39)	20 (9.35)			154 (9.22)	111 (8.89)	43 (10.19)		
Vegetable grease	1,046 (91.43)	852 (91.61)	194 (90.65)			1,516 (90.78)	1,137 (91.11)	379 (89.81)		
Taste preference, *n* (%)				*χ*^2^ = 3.23	0.072				*χ*^2^ = 4.08	0.043
Other	389 (34.00)	305 (32.80)	84 (39.25)			459 (27.49)	327 (26.20)	132 (31.28)		
Light taste	755 (66.00)	625 (67.20)	130 (60.75)			1,211 (72.51)	921 (73.80)	290 (68.72)		
Co-residence, *n* (%)				*χ*^2^ = 0.48	0.487				*χ*^2^ = 0.95	0.329
Alone	167 (14.60)	139 (14.95)	28 (13.08)			328 (19.64)	252 (20.19)	76 (18.01)		
Other	977 (85.40)	791 (85.05)	186 (86.92)			1,342 (80.36)	996 (79.81)	346 (81.99)		
Residence, *n* (%)				*χ*^2^ = 0.83	0.363				*χ*^2^ = 0.41	0.52
Urban	556 (48.60)	446 (47.96)	110 (51.40)			822 (49.22)	620 (49.68)	202 (47.87)		
Town	588 (51.40)	484 (52.04)	104 (48.60)			848 (50.78)	628 (50.32)	220 (52.13)		
Marital status, *n* (%)				*χ*^2^ = 2.09	0.148				*χ*^2^ = 3.73	0.053
Other	416 (36.36)	329 (35.38)	87 (40.65)			1,095 (65.57)	802 (64.26)	293 (69.43)		
Married	728 (63.64)	601 (64.62)	127 (59.35)			575 (34.43)	446 (35.74)	129 (30.57)		
Education level, *n* (%)				*χ*^2^ = 6.42	0.04				*χ*^2^ = 5.34	0.069
Illiteracy	288 (25.17)	220 (23.66)	68 (31.78)			1,083 (64.85)	792 (63.46)	291 (68.96)		
Primary school or below	592 (51.75)	488 (52.47)	104 (48.60)			468 (28.02)	359 (28.77)	109 (25.83)		
Secondary school or above	264 (23.08)	222 (23.87)	42 (19.63)			119 (7.13)	97 (7.77)	22 (5.21)		
Insurance, *n* (%)				*χ*^2^ = 0.01	0.943				*χ*^2^ = 0.07	0.797
No	639 (55.86)	519 (55.81)	120 (56.07)			1,032 (61.80)	769 (61.62)	263 (62.32)		
Yes	505 (44.14)	411 (44.19)	94 (43.93)			638 (38.20)	479 (38.38)	159 (37.68)		

*χ2, Chi-square test; BMI, body mass index; IADL, instrumental activity of daily living; BADL, basic activity of daily living.*

The results of the binary logistic regression analysis shown in Table 4 indicated that in men, IADL disability, fresh fruit, and taste were important associated factors for falls among older adults with hypertension. Specifically, people with IADL disability (OR = 1.585; 95% CI: 1.063–2.362) were more likely to fall than normal people, those who ate fresh fruit (OR = 0.640; 95% CI: 0.457 to 0.895) were less likely to fall than those who did not, and those with a light taste (OR = 0.712; 95% CI: 0.513–0.989) had a lower risk of falling than people with other tastes. In women, self-rated health, stroke, IADL disability, exercise, and taste preference were important factors for falls among older adults with hypertension. Those with self-rated poor health (OR = 1.446; 95% CI: 1.115–1.876), those with stroke (OR = 1.423; 95% CI: 1.022–1.981), and those with IADL disability (OR = 1.617; 95% CI: 1.168–2.237) were more likely to fall than normal people. Also, people who exercised regularly (OR = 1.410; 95% CI: 1.077–1.847) were more likely to fall than those who did not. In contrast, people with a light taste (OR = 0.768; 95% CI: 0.5999–0.985) were more likely to have a lower risk of falls than those with other tastes.

**Table 4:** Logistic regression results of the falls in male and female.

Variables	Male			Female			Coefficient(B)
	OR	95% CI	*p*	OR	95% CI	*p*	
Age, *n* (%)							
60–79	1.00 (Ref.)	1.00 (Ref.)		1.00 (Ref.)	1.00 (Ref.)		
≥ 80	0.894	0.601–1.330	0.580	0.949	0.684–1.317	0.754	0.030
BMI, *n* (%)							
< 18.5	1.00 (Ref.)	1.00 (Ref.)	0.091	1.00 (Ref.)	1.00 (Ref.)		
18.5–24	1.564	0.883–2.767	0.125	1.071	0.743–1.543	0.714	0.345
24–28	1.117	0.599–2.082	0.728	0.879	0.575–1.344	0.551	0.201
≥ 28	0.894	0.414–1.929	0.775	0.912	0.548–1.517	0.722	0.034
Abdominal obesity, *n* (%)							
No	1.00 (Ref.)	1.00 (Ref.)		1.00 (Ref.)	1.00 (Ref.)		
Yes	1.220	0.667–2.231	0.520	0.987	0.757–1.285	0.921	0.089
Self-rated health, *n* (%)							
Good	1.00 (Ref.)	1.00 (Ref.)		1.00 (Ref.)	1.00 (Ref.)		
Bad	1.235	0.865–1.762	0.245	1.446	1.115–1.876	0.005	0.062
Hearing impairment, *n* (%)							
No	1.00 (Ref.)	1.00 (Ref.)		1.00 (Ref.)	1.00 (Ref.)		
Yes	1.342	0.949–1.896	0.096	1.255	0.962–1.638	0.094	0.112
Visual impairment, *n* (%)							
No	1.00 (Ref.)	1.00 (Ref.)		1.00 (Ref.)	1.00 (Ref.)		
Yes	0.996	0.695–1.427	0.982	0.930	0.718–1.205	0.585	0.149
Heart disease, *n* (%)							
No	1.00 (Ref.)	1.00 (Ref.)		1.00 (Ref.)	1.00 (Ref.)		
Yes	1.304	0.875–1.944	0.193	1.070	0.809–1.414	0.637	0.188
Diabetes, *n* (%)							
No	1.00 (Ref.)	1.00 (Ref.)		1.00 (Ref.)	1.00 (Ref.)		
Yes	1.328	0.851–2.070	0.211	0.958	0.680–1.350	0.808	0.306
Stroke, *n* (%)							
No	1.00 (Ref.)	1.00 (Ref.)		1.00 (Ref.)	1.00 (Ref.)		
Yes	1.367	0.910–2.053	0.132	1.423	1.022–1.981	0.037	−0.003
BADL disability, *n* (%)							
No	1.00 (Ref.)	1.00 (Ref.)		1.00 (Ref.)	1.00 (Ref.)		
Yes	1.516	0.980–2.346	0.061	1.324	0.983–1.783	0.065	0.200
IADL disability, *n* (%)							
No	1.00 (Ref.)	1.00 (Ref.)		1.00 (Ref.)	1.00 (Ref.)		
Yes	1.585	1.063–2.362	0.024	1.617	1.168–2.237	0.004	0.105
Exercise, *n* (%)							
No	1.00 (Ref.)	1.00 (Ref.)		1.00 (Ref.)	1.00 (Ref.)		
Yes	1.111	0.788–1.566	0.548	1.410	1.077–1.847	0.013	−0.305*
Smoking, *n* (%)							
No	1.00 (Ref.)	1.00 (Ref.)		1.00 (Ref.)	1.00 (Ref.)		
Yes	0.841	0.581–1.218	0.360	0.690	0.372–1.279	0.239	0.254
Drinking, *n* (%)							
No	1.00 (Ref.)	1.00 (Ref.)		1.00 (Ref.)	1.00 (Ref.)		
Yes	1.263	0.863–1.848	0.230	1.069	0.628–1.820	0.805	0.163
Self-reported quality of life, *n* (%)							
Good	1.00 (Ref.)	1.00 (Ref.)		1.00 (Ref.)	1.00 (Ref.)		
Bad	1.407	0.990–2.001	0.057	0.942	0.720–1.233	0.666	0.359
Fresh fruit, *n* (%)							
No	1.00 (Ref.)	1.00 (Ref.)		1.00 (Ref.)	1.00 (Ref.)		
Yes	0.640	0.457–0.895	0.009	0.867	0.669–1.123	0.279	−0.356*
Grease, *n* (%)							
Animal grease	1.00 (Ref.)	1.00 (Ref.)		1.00 (Ref.)	1.00 (Ref.)		
Vegetable grease	0.857	0.496–1.480	0.580	0.795	0.540–1.170	0.244	0.081
Taste preference, *n* (%)							
Other	1.00 (Ref.)	1.00 (Ref.)		1.00 (Ref.)	1.00 (Ref.)		
Light taste	0.712	0.513–0.989	0.043	0.768	0.599–0.985	0.038	−0.092
Co-residence, *n* (%)							
Alone	1.00 (Ref.)	1.00 (Ref.)		1.00 (Ref.)	1.00 (Ref.)		
Other	1.349	0.813–2.237	0.246	1.075	0.786–1.470	0.652	0.156
Residence, *n* (%)							
Urban	1.00 (Ref.)	1.00 (Ref.)		1.00 (Ref.)	1.00 (Ref.)		
Rural	0.853	0.622–1.169	0.323	1.039	0.824–1.309	0.746	−0.210
Marital status, *n* (%)							
Other	1.00 (Ref.)	1.00 (Ref.)		1.00 (Ref.)	1.00 (Ref.)		
Married	0.873	0.595–1.281	0.489	0.959	0.711–1.293	0.782	−0.031
Education level, *n* (%)							
Illiteracy	1.00 (Ref.)	1.00 (Ref.)	0.636	1.00 (Ref.)	1.00 (Ref.)		
Primary school or below	0.837	0.576–1.217	0.352	0.976	0.734–1.300	0.870	−0.245
Secondary school or above	0.845	0.518–1.377	0.499	0.716	0.419–1.222	0.220	0.064
Insurance, *n* (%)							
No	1.00 (Ref.)	1.00 (Ref.)		1.00 (Ref.)	1.00 (Ref.)		
Yes	0.988	0.720–1.357	0.941	0.968	0.764–1.227	0.790	−0.003

BMI, body mass index; IADL, instrumental activity of daily living; BADL, basic activity of daily living; Ref., reference.

The results of the interactive analysis in Table 4 showed that exercise (coefficient = −0.305, *p* < 0.05) and fresh fruit (coefficient = −0.356, *p* < 0.05) had statistically significant interaction with gender, suggesting that there were significant gender differences in the effects of these two variables on falls.

## 4 Discussion

Based on the nationally representative survey data, we observed that 22.60% of older adults with hypertension experienced falls. Logistic regression results suggested that people with poor self-rated health, hearing impairment, stroke, BADL disability, IADL disability, and regular exercise were more likely to fall, while men, people who ate fresh fruit, and people with a light taste were less likely to fall. In addition, our analysis results showed that there were significant gender differences in the effects of exercise and fresh fruit on falls.

Our study found that older women with hypertension had a higher prevalence of falls (25.27%) than men (18.71%). Multivariate logistic regression analysis revealed that the fall risk of older men with hypertension was 0.766 times that of the women. These results are consistent with some previous studies showing that there were gender differences in the risk of falls among older adults (12, 22). One possible explanation is that older Chinese women are more likely to suffer from osteoporosis, which affects their bone and muscle function, making their knee muscles weaker (23), and thus increasing the risk of falls. A lack of estrogen in women’s bodies after menopause may cause bone loss in women at a faster rate than in men, reducing their physical function (24, 25).

This study found that people with poor self-rated health were more likely to fall than those with good self-rated health. Previous studies have shown that falls are associated with poorer self-rated health among older Chinese adults (26, 27). Physically, people with poorer self-rated health are more likely to have chronic diseases, such as diabetes (28). Chronic diseases can lead to sarcopenia, which increases the risk of falls in older people (29). Psychologically, poor self-rated health is an important predictor of depression (30). Studies have shown that depressive symptoms can lead to psychomotor disorders, which can impair an individual’s balance and ability to effectively respond to environmental challenges and increase the risk of falls (31). People with depression often have abnormal standing posture, which can result in falls (31). There are also some studies suggesting that side effects of medications used to treat depressive symptoms can also increase the risk of falls (31). It is worth mentioning that our findings found that self-rated health only had a significant impact on older women with hypertension, which may be because women tend to be more sensitive to their own health status, and when the self-rated health status is poor, they may have greater psychological stress and anxiety, which will interfere with the nervous system’s control of muscles and affect body coordination and reaction speed, thereby increasing the risk of falls.

Consistent with previous findings, older adults with hearing impairment were 2.4 times more likely to have a fall than those with normal hearing of the same age and were more likely to have a fatal injury (32, 33). This may be because the hearing system is closely related to maintaining balance in the body. Studies have shown that the auditory system is responsible for integrating head and body movement and position information into the brainstem, cerebellum, and somatosensory cortex to maintain balance (34). Damage to the hearing system affects the vestibular system, causing many people with age-related hearing loss to experience dizziness, resulting in an imbalance in the body (35, 36). At the same time, hearing impairment reduces perception, resulting in an increased burden on cognitive and attention resources that are also necessary for maintaining bodily functions such as postural control and balance (36, 37). Part of the explanation for the association between hearing impairment and fall risk could also be that older adults with hearing impairment are unable to hear their own footfall (38). In our findings, hearing impairment had a significant effect on falls across the population, but no significant associations were identified in subgroup analyses of men and women. This may be due to the small sample size of males and females in the subgroup analysis, resulting in insufficient statistical power. Therefore, the sample size should be further expanded in future studies to determine the causes of this difference.

Our results also demonstrated that people who had stroke were more likely to have falls than those who did not. Falls are a common complication after stroke, and both the physical and psychological impairments associated with stroke can lead to frequent falls later in life (39). Physically, residual sensorimotor deficits after stroke can cause impaired balance and increase the risk of falls among older adults (40). At the same time, stroke survivors are prone to osteoporosis (41). Accelerated bone loss often leads to an increased risk of fracture in stroke patients, and further increases the risk of falls (42), and fractures resulting from falls can affect the patient’s mobility, creating a vicious cycle (39). In terms of psychology, studies have revealed that the reason for the increased risk of falls in older stroke patients may be a maladaptive fear of falls caused by factors such as depression, anxiety, past fall experiences, poor balance, and limited mobility, and this maladaptive fear of falls can cause older adults to limit their social activities, resulting in muscle atrophy and decreased athletic ability (43). Of concern, stroke only had a significant effect on falls in older women with hypertension, possibly because women are more likely than men to have osteoporosis, have less muscle strength and are therefore more likely to have falls during stroke recovery (23).

People with BADL and IADL disability were more likely to fall than normal people. Studies have shown that people with BADL and IADL disability are more likely to have limited mobility and slow gait, which affects body balance (23, 25, 44). People with BADL and IADL disability have worse self-care ability (21), and being less involved or not participating in social activities for a long time not only leads to muscle atrophy and continuous decline in exercise ability (43) but also increases their loneliness and thus increases the risk of depression (45). At the same time, poor social relationships can give rise to reduced access to health care and reduced adherence to medication (25). We found that BADL disability had no significant effect on falls in subgroup analyses of both older male and female patients, with possible causes consistent with the ‘hearing impairment’ explanation.

Taste preference had a significant effect on falls among older adults with hypertension, both in the population as a whole and in men and women, respectively. That is, people with a light taste were less likely to fall than people with other tastes. The light taste helps patients control the occurrence and progression of chronic diseases such as high blood pressure and reduces patients’ dependence on medication (46). For example, a light diet is low in sodium, and there is a broad linear association between sodium intake and the risk of complex cardiovascular outcomes (stroke, myocardial infarction, coronary revascularization) (47, 48). At the same time, a light diet helps to maintain a healthy weight and reduce the burden on the body, thereby improving the flexibility and balance of the body.

Fresh fruit was also a significant risk factor in our study. In addition to containing micronutrients and macronutrients, fresh fruit also contains a large amount of bioactive compounds (49). Bioactive compounds are compounds present in foods that produce reproducible biological effects at the dietary level, with polyphenols as a representative (50). Polyphenols contain flavonoids, which have a variety of biological activities such as anti-inflammatory, antioxidant, anti-free radical and neuroprotective effects (51). Long-term intake of polyphenols can not only reduce oxidative stress in muscles and have a positive impact on mitochondria, but also can beneficially act on blood vessels, ensure the supply of nutrients to muscles, and prevent loss of muscle mass (52). Therefore, eating fresh fruit can improve muscle strength and balance, and reduce the risk of falls (50). At the same time, eating fresh fruit can also prevent and control high blood pressure and other chronic diseases (49, 53).

Interestingly, our results suggested that people who exercised regularly were more likely to fall than those who did not, contrary to most previous research. Previous studies have shown that exercise can reduce the risk of falls by improving balance and leg strength among older adults (54–56). At the same time, a small number of studies have shown that exercise has no effect on preventing falls (57, 58). The differences in these findings may be due to differences in exercise styles. Studies have shown that long-term exercises, especially those that promote balance, can reduce the risk of falls, but short-term exercises, more complex exercises, or exercises that do not include balance and function training do not reduce the risk of falls (59, 60). In fact, this difference is still not fully explained by research, and future studies could employ more refined experimental designs to reveal the complex dynamics behind it.

In addition, the results of the interaction analysis showed that there was a significant interaction between exercise and fresh fruit on the impact of falls in hypertensive patients. Our findings suggest that exercise is a significant risk factor for falls only in older women with hypertension. Influenced by exercise habits and roles, women may be less willing to participate in physical exercise than men. At the same time, there are differences in the ways of exercise among older adults of different genders, which may contribute to gender differences affecting falls. In addition, the body structures of men and women are different, men’s fat is mainly stored in the abdomen, and women’s fat is mainly stored in the buttocks and hips (61), which may have an impact on the effect of exercise.

The effect of fresh fruit on falls among older adults with hypertension was significant only in men. Women’s diets are typically characterized by higher carbohydrate intake, including fruit and vegetables. In contrast, men consume more of an animal protein-rich diet and less fruit than women (62). Research results show that women consume more fruit than men (2.6 servings/day vs. 2.2 servings/day) (63). Therefore, the potential changes in the body caused by eating fresh fruit may be more significant in men than in women.

## 5 Conclusion

Overall, falls are common among older adults with hypertension, with a prevalence of 22.60%. Based on the five dimensions of the Health Ecology Model, the Logistic Regression Model identified gender, self-rated health, hearing impairment, stroke, BADL disability, IADL disability, exercise, fresh fruit and taste preference as significant associated factors for falls among older adults with hypertension. Among them, the effects of self-rated health, stroke and exercise on falls were only significant in female hypertensive patients. The effect of fresh fruit on falls was significant only in men with hypertension. The results of further interaction analysis showed that there were significant gender differences in the effects of exercise and fresh fruit on falls. These results suggest that in the clinical practice of focusing on falls in hypertensive patients, it may be effective to target these significant influencing factors and carry out appropriate interventions.

## 6 Limitations

Admittedly, there are still some limitations to our study. First, this study used cross-sectional data published by the CLHLS database in 2018, unable to establish a causal relationship between the fall risk of older adults with hypertension and their associated factors, which is worthy of further proof in future cohort studies. Second, the measurement of all indicators mainly relied on the self-reported data of the participants, and although the investigators were professionally trained, there were still some inevitable subjective biases. Third, although we applied the HEM to conduct a relatively comprehensive examination of associated factors, due to the limitation of investigation resources in the database, fewer factors were included in the living and working conditions and policy environment dimensions, and other potentially significant important variables (such as pre-retirement occupation and public health policy) were not included in the study. Finally, although the data in our study came from a high-quality nationwide survey, the sample size obtained is still limited due to the limitations of special populations and the absence of some data. It is suggested that further studies with larger sample sizes should be conducted to verify these conclusions.

## Data Availability

The datasets presented in this study can be found in online repositories. The names of the repository/repositories and accession number(s) can be found in the article/Supplementary material.

## Footnotes

^1^ https://opendata.pku.edu.cn/dataverse/pku/
