# Supplementary material for: Associated factors and gender differences of falls in older adults with hypertension: a national cross-sectional survey
Source: Front Public Health. 2025 Apr 16;13:1537587. doi: 10.3389/fpubh.2025.1537587 (PMC12040895; doi:10.3389/fpubh.2025.1537587)
Supplement: Supplementary file 1 [file Table_1.docx]

**Appendix 1:Questionnaire Items and Classification Criteria of Covariates**

| Variables | | Questionnaire Items | Classification assignment | |
| --- | --- | --- | --- | --- |
| Individual level | Gender | Sex | Female=0 | Male=1 |
|  | Age | Validated age | 60-79=0 | ≥80=1 |
|  | BMI | Weight (kilograms) & height (Centimeter) | <18.5 =0 | 18.5-24 =1 |
|  |  |  | 24-28 =2 | ≥28 =3 |
|  | Abdominal obesity | Waist circumference | No=0 | Yes=1 |
|  | Self-rated health | How do you rate your health at present? | Good=0 | Bad=1 |
|  | Hearing impairment | Do you have any difficulty with your hearing? | No=0 | Yes=1 |
|  | Visual impairment | Visual function: can you see the break in the circle? | No=0 | Yes=1 |
|  | Diabetes | Suffering from diabetes? | No=0 | Yes=1 |
|  | Heart disease | Suffering from heart disease? | No=0 | Yes=1 |
|  | Stroke | Suffering from stroke or cvd? | No=0 | Yes=1 |
|  | IADLs: Unable to visit neighbors by oneself | Can you visit your neighbors by yourself? | No=0 | Yes=1 |
|  | IADLs: Unable to shop by oneself if necessary | Can you go shopping by yourself? | No=0 | Yes=1 |
|  | IADLs: Unable to cook meals by oneself if necessary | Can you cook a meal by yourself whenever necessary? | No=0 | Yes=1 |
|  | IADLs: Unable to wash clothing by oneself | Can you wash clothing by yourself whenever necessary? | No=0 | Yes=1 |
|  | IADLs: Unable to walk continuously for 1 kilometer | Can you walk continuously for 1 kilometer at a time by yourself? | No=0 | Yes=1 |
|  | IADLs: Unable to lift a weight of 5 kg | Can you lift a weight of 5kg, such as a heavy bag of groceries? | No=0 | Yes=1 |
|  | IADLs: Unable to continuously crouch and stand up three times | Can you continuously crouch and stand up three times? | No=0 | Yes=1 |
|  | IADLs: Unable to use public transportation | Can you take public transportation by yourself? | No=0 | Yes=1 |
|  | BADLs: Needs assistance bathing | Bathing–either sponge bath, tub bath, shower, or washing the body | No=0 | Yes=1 |
|  | BADLs: Needs assistance dressing | Dressing–gets clothes from closets and drawers | No=0 | Yes=1 |
|  | BADLs: Needs assistance toileting | Toilet–going to the toilet; cleaning oneself afterward | No=0 | Yes=1 |
|  | BADLs: Needs assistance going in indoor | Indoor Transfer | No=0 | Yes=1 |
|  | BADLs: Needs assistance eating | Continence | No=0 | Yes=1 |
|  | BADLs: Needs Incontinence | Eating | No=0 | Yes=1 |
| Behavior level | Exercise | Do you do exercises regularly at present? | No=0 | Yes=1 |
|  | Smoking | Do you smoke at the present time? | No=0 | Yes=1 |
|  | Drinking | Do you drink alcohol at the present time? | No=0 | Yes=1 |
|  | Self-reported quality of life | How do you rate your life at present? | Good=0 | Bad=1 |
|  | Fresh fruit | Do you eat fresh fruit? | No=0 | Yes=1 |
|  | Grease | What kind of grease do you mainly use for cooking? | Animal grease=0 | Vegetable grease=1 |
|  | Taste preference | What kind of flavor do you mainly have? | Other=0 | Light taste=1 |
| Interpersonal level | Co-residence | Co-residence | Alone =0 | Other =1 |
|  | Residence | Current residence area of the interviewee | Urban =0 | Rural=1 |
|  | Marital status | Current marital status | Other =0 | Married=1 |
| Life and work conditions level | Education level | How many years did you attend school? | Illiteracy =0 | |
|  |  |  | Primary school or below =1 | |
|  |  |  | Secondary school or above=2 | |
| Policy environment level | Insurance | Do you participate in public old-age insurance? | No=0 | Yes=1 |

Note: BMI: body mass index; IADLs: instrumental activities of daily living; BADLs: basic activities of daily living
